# Effects of Sociality Level on Companion Dog Training through Food Reinforcement

**DOI:** 10.3390/ani10122413

**Published:** 2020-12-17

**Authors:** Ok-Deuk Kang

**Affiliations:** Animal Technology, College of Applied Life Science, Jeju National University, 102 Jejudaehak-ro, Jeju-si 63243, Korea; kod0816@gmail.com; Tel.: +82-64-727-7005

**Keywords:** dog behavior, dog training, food reinforcement, interaction, sociability, welfare

## Abstract

**Simple Summary:**

This test evaluates how your dog reacts to strangers’ behavior. Dogs with high sociality interact positively with strangers, such as physical contact or close proximity to strangers. On the other hand, dogs with low social levels may exhibit fear and fear-related behaviors or may show aggressiveness to relieve them. Highly social dogs tend to keep their eyes on their owners longer and are more likely to interact with humans. This study showed that the results of social training of dogs through food enrichment were much more effective in both the high sociality and low sociality groups. In particular, the high sociality group showed a much higher increase in social behavior than the low sociality group. This suggests that sociality is highly related even in the process of communication and training between dogs and humans. It also shows that it can be reinforced more effectively in dog-human interactions.

**Abstract:**

The purpose of this study was to investigate the effect of sociality level in dogs using food reinforcement. The companion dogs living mostly inside (IS) and those living mostly outside (OS) groups were further classified into high sociality (HS) and low sociality (LS) groups using a social test. The data were analyzed by observing videos of the dogs and recording nine categories of sociality. Passive; activity; and communication tasks were measured in terms of the time the dog first contacted the trainer; the time it took for the dog to approach within 1 m of the trainer, and the time the dog remained in contact. The IS and OS groups showed no significant differences in any category; except for close to trainer within 1 m of the active phase. However, in a comparison between the overall HS and LS groups; significant positive changes were observed in all items except for the first contact time to the trainer of the passive and active phase. This is an important result of the sociality level; as positive results can be predicted not only in a dog’s ability to adapt to the environment but also in the communication and training exchanges between dogs and humans

## 1. Introduction

Throughout human history, dogs have been faithful companions and protectors. Sociality is an essential factor for dogs in establishing close relationships with humans [1]. The sociality of dogs refers to the tendency of dogs to maintain a healthy social life, such as communication with humans and other animals and conveying emotions in the process of their growth and development. In modern society, dogs have many working roles, such as hunting and herding [2], but mostly they are companions and members of the family [3,4]. As such, the relationship between humans and dogs is important for healthy and happy coexistence and contributes positively to the interaction based on high sociality [5]. Dogs with high sociality can communicate with each other through training [6], adapt well to changing situations and environments [7], and have positive interactions with strangers [6].

Dog sociality, which can be created via a short socialization period in the puppy stage, is maintained throughout the dog’s life [8]; thus, their development from an early age is emphasized as being especially beneficial as dogs adapt to life as a family companion [7]. However, some dogs tend to be more sensitive and prone to stress with respect to strangers, dogs, and other animals, and unfamiliar places [9]. In particular, this stress can cause serious problems with family life [10] and have a negative effect on the human–dog relationship [11]. For example, fearful dogs tend to show fear-related aggression toward other dogs [12]. Additionally, the lower the social level, the higher the aggression toward strangers [13].

Behavioral assessment is used mainly for social assessment in dogs [6,7,14,15]. Among various techniques, the most commonly used method for sociality evaluation is the Ainsworth Strange Situation Test [16], which has since been partially modified [6,14,17]. This test evaluates how the dog reacts to the appearance of a stranger. Dogs with high sociality interact positively, such as via physical contact or remaining in close proximity to strangers [5]. On the other hand, dogs with low sociality may exhibit fear-related behaviors or aggressiveness [13]. Therefore, dogs need to be supported by basic training to maintain a smooth life with humans. In addition, training methods can be an important issue for their well-being [18].

In general, the training method of an animal refers to a type of learning that is learned when an animal behaves in an environment and is mainly implemented based on operant conditioning [19] Operant conditioning refers to a method of increasing or decreasing the frequency at which the behavior occurs through reinforcement and punishment for a dog’s reaction. In this process, reinforcement plays an important role. There are two types of reinforcement: positive reinforcement, which increases the frequency of desired actions by providing preferred stimuli to dogs, and negative reinforcement, which increases the frequency of actions through the removal of hate stimuli.

In a previous study on dog training methods, Deldalle and Gaunet [18] found that positive and negative reinforcement studies show that the positive reinforcement training method is less stressful and more effective in dogs’ welfare, Hiby et al. [20] and Black et al. [21] also reported that the positive reinforcement method has fewer behavioral problems and higher training effects. Therefore, the learning task of this study focused on the reinforcement method through food reward (positive reinforcement). It is hypothesized that this method will be applied rapidly in human and dog interactions and that it will be directly related to the effectiveness of training.

Recently, the number of dogs in Korea is increasing very much [22], However, despite the rapid increase in the domestic dog population, there have been few studies on the sociality of dogs.

Additionally, as a member of the household, the dog may reside inside the house or stay outside in the yard. The purpose of this study was to evaluate the sociability of the dog in performing the “sit” and “wait” commands from a trainer based on a food reinforcement method; a comparison was made between dogs that lived mostly inside the home and those that spent most of their time outside in the yard.

## 2. Materials and Methods

### 2.1. Experimental Animals

For this study, the dog participants were identified via a dog education network, after obtaining consent from the dogs’ owners. Of these, 28 dogs with no previous special training were included and then divided into two groups according to whether the dog lived mainly indoors (IS group) or outdoors (OS group). Also, via the sociality evaluation, the dogs from each group were further classified as having a high or low level of sociality (HS and LS groups, respectively); the details of the individual groups are listed in Table 1. The IS (n = 14; 4.6 ± 3.13 years) and OS (n = 14; 3.01 ± 1.25 years) groups were each divided into HS (n = 7 [5.40 ± 4.30 years; 4 male castrated and 3 female spayed] and n = 8 [3.80 ± 1.07 years; 3 male castrated, 3 female spayed, and 2 females], respectively) and LS (n = 7 [3.11 ± 1.57 years; 2 male castrated, 3 female spayed, and 2 males, 1 female] and n = 6 [2.87 ± 0.73 years; 3 male castrated, 1 male, and 2 females], respectively) groups. This study was approved by the Institutional Animal Care and Use Committee of Jeju National University, South Korea (Approval No. 2020-0031). To reduce the stress of the dogs, the experimental site for the social test was performed at a location in close proximity to the study participants, and the communication behavior task was performed at a local outdoor training facility.

### 2.2. Experimental Procedure

#### 2.2.1. Sociality Test

**Figure 1:**
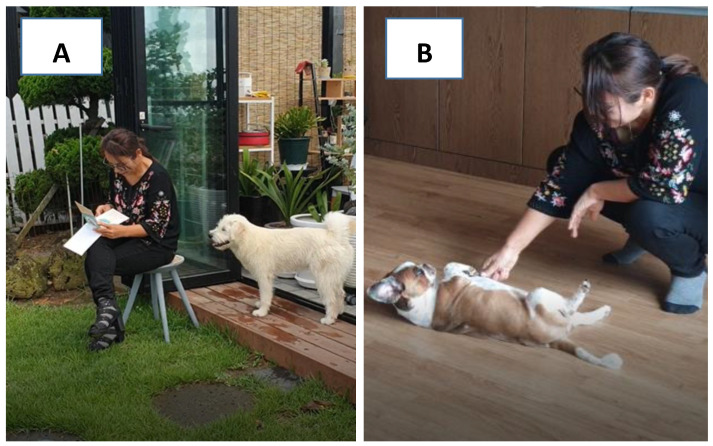
(**A**) Evaluation of the passive phase for sociality test (Step 1) and (**B**) Evaluation of the active phase for sociality test (Step 2).

The sociality test was conducted to assess the dog’s initial reaction to strangers and to classify the dog’s social assessment level. An experimenter who had never seen the dogs initiated the test. At the test site, a chair was placed where the experimenter would sit, and a narrow tape (so that the dog would not be uncomfortable) was attached to the floor at a distance of 1 m around the chair to gauge the distance as the dog approached the experimenter (Figure 1). To differentiate between HS and LS dogs and to accurately evaluate the level of sociality, a behavioral test was performed based on the protocol presented by Batt et al. [23], Barrera et al. [14], and Jakovcevic et al. [6]. At the experiment site, a chair for the experimenter to sit was placed on the other side of a door. Dogs were given 5 min to adjust to the test site with their owner.

The steps of the evaluation are given below.

Step 1: Evaluation of the passive phase was conducted for 2 min, starting when the experimenter entered and sat on a chair, and the owner left the room. During this process, the experimenter made sure not to attempt any contact (including visual contact) with the dog at first. However, once the dog made physical contact, the dog’s head or chin was patted for 3 to 5 s. This response from the experimenter was repeated each time the dog made contact (Figure 1A).

Step 2: In the evaluation of the active phase (for 2 min), the experimenter arose from the chair and sang gently while making eye contact with the dog. In this process, the experimenter asked the dog to make contact first. When the dog approached, the experimenter spoke and stroked the dog’s head. If the dog did not come, the experimenter called the dog’s name (10 s intervals, three times), in an attempt to get the dog to approach (Figure 1B).

#### 2.2.2. Social Evaluation Items

The level of sociability was classified via a behavioral evaluation test, and evaluation items in six categories were analyzed [6,14]. In addition, there were three categories of communication behavior tasks. Thus, a total of nine categories were evaluated. The variables in the passive phase were the time the dog first contacted the trainer (PCL), the time the dog approached the trainer within 1 m (PTC), and the time the dog remaining in physical contact with the trainer (PPC). The duration before the first contact with the trainer (ACL), the duration it took for the dog to approach the trainer within 1 m (ATC), and the duration of physical contact with the trainer (APC) were also examined (Table 1).

#### 2.2.3. Communication Behavior Task

The action task was to train the dog to “sit” and “wait”. The dog was required to sit when the trainer asked it to “sit” while moving and holding the dog’s leash. In addition, the goal was to wait at least 5 s when the trainer asked the dog to “wait”. The communication behavior test was carried out at an outdoor training ground (dimensions: 10 × 4 m). The owner and dog were allowed approximately 10 min to explore the test site before the test began. During this time, the trainer was located next to the dog’s shoulder girdle and did not talk or take any action. The owner of the dog undergoing the test then placed the lead strap down on the floor and left. For 5 min, the trainer observed the dog’s reaction while calling the dog’s name three times without moving.

Period 1 (warm-up, 3 min): the trainer actively called the dogs. When the dog looked at the trainer, it was given food (frozen pig liver) fortification three or four times; notably, the food was presented to the dog in the palm of the trainer’s hand to encourage the dog to approach and eat. After that, the food reward was given only when the dog performed the “sit” command.

Period 2 (training, 5 min): when the trainer commanded the dog to “sit” while moving and holding the lead line, the goal was for the dog to sit down and perform the “wait” behavior on command. To achieve the goal, the trainer grabbed the leash and moved around the training ground, instructing the dog to “sit down” and “wait”. Each time the dog responded correctly to the order, the dog was rewarded with an audible “good job” from the trainer.

Period 3 (evaluation, 2 min): the trainer was different from the trainer in Period 2. The trainer stood next to the snack container and called the dog only once. Food reinforcement was given when a dog came and met his gaze and ordered “sit” and “wait”.

“Sit” and “wait” orders were given up to three times (10 s intervals). The assessment assessed the dog’s initial contact with the trainer (RCL), close proximity (RTC), or time to maintain contact (RPC).

### 2.3. Data Analysis

Data collection involved observing videos and recording the nine categories of sociality. SPSS version 24.0 (IBM Corp., Armonk, NY, USA) was used for the statistical analysis. Differences between groups were analyzed using the nonparametric Mann–Whitney U test, and one-way analysis of variance was used for comparisons by stage, and Duncan’s test was used as a post hoc test. The significance level was set to *p* < 0.05.

## 3. Results

As a result of comparing the high social group with the low social group, there were significant behavioral differences in dogs related to access to the trainer, and the high social group showed higher accessibility. The results of the nine categories of the social behavior test are shown in Table 2. There was no significant difference between the IS and OS groups in any of the nine categories except for close to trainer within 1 m of the active phase. However, in a comparison between the overall HS and LS groups, close to trainer within 1m of passive phase (*p* < 0.01), physical contact with the trainer of passive phase (*p* < 0.001), close to trainer within 1 m of active phase (*p* < 0.5), physical contact with the trainer of active phase (*p* < 0.001), first contact time to trainer of food reinforcement phase (*p* < 0.5), close to trainer within 1 m of food reinforcement phase (*p* < 0.5), and RPC physical contact with the trainer of food reinforcement phase (*p* < 0.001) were significantly different. The HS and LS groups within the IS group differed significantly with respect to physical contact with the trainer of passive phase (*p* < 0.5), physical contact with the trainer of active phase (*p* < 0.001), and physical contact with the trainer of food reinforcement phase (*p* < 0.001). There was a significant difference between the HS and LS groups within the OS group regarding close to trainer within 1 m of passive phase (*p* < 0.5), physical contact with the trainer of passive phase (*p* < 0.001), physical contact with the trainer of active phase (*p* < 0.001), first contact time to trainer of food reinforcement phase (*p* < 0.01), close to trainer within 1 m of food reinforcement phase (*p* < 0.01), and physical contact with the trainer of food reinforcement phase (*p* < 0.001) (Table 2).

**Figure 2:**
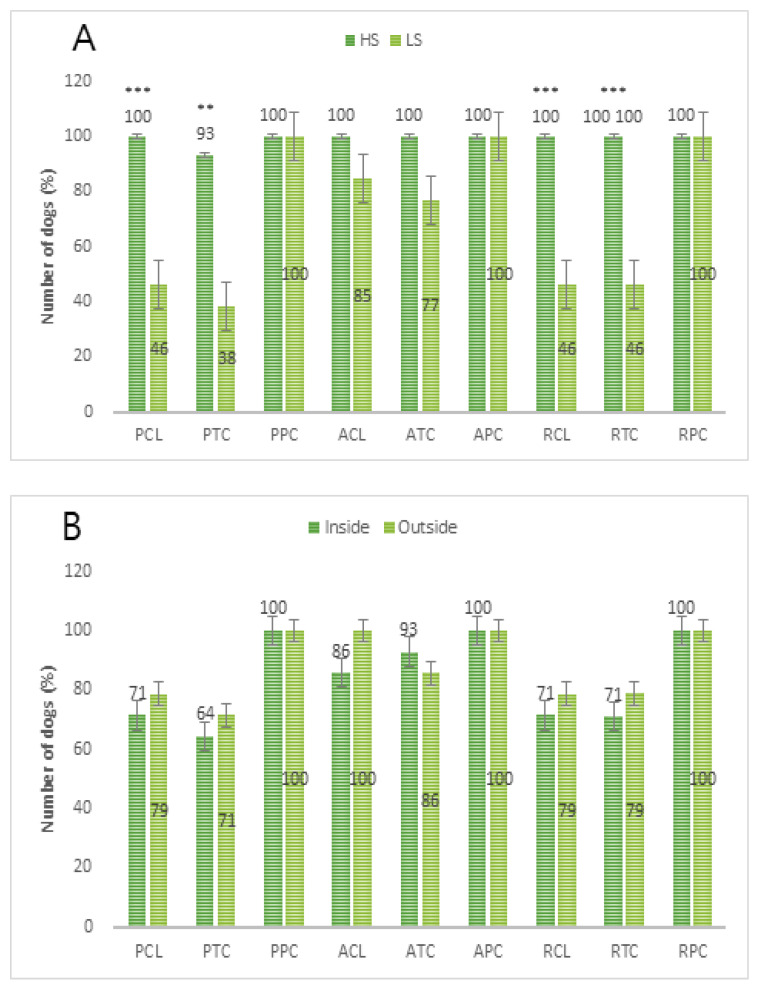
Behavioral changes in dogs in the 9 social behavior test categories. (**A**): Comparison between high sociality (HS) group and low sociality (LS) group, Levels of significance: ** *p*< 0.01; *** *p*< 0.001. (**B**): Comparison between the living mostly inside (IS) group and the living mostly outside (OS) group. PCL, contact latency of passive phase; PTC, time close to the trainer of passive phase; PPC, physical contact time of passive phase; ACL, contact latency of active phase; ATC, time close to the trainer of active phase; APC, physical contact with the trainer of active phase; RCL, contact latency of food reinforcement phase; RTC, time close to the trainer of food reinforcement phase; RPC, physical contact with the trainer of food reinforcement phase.

**Figure 3:**
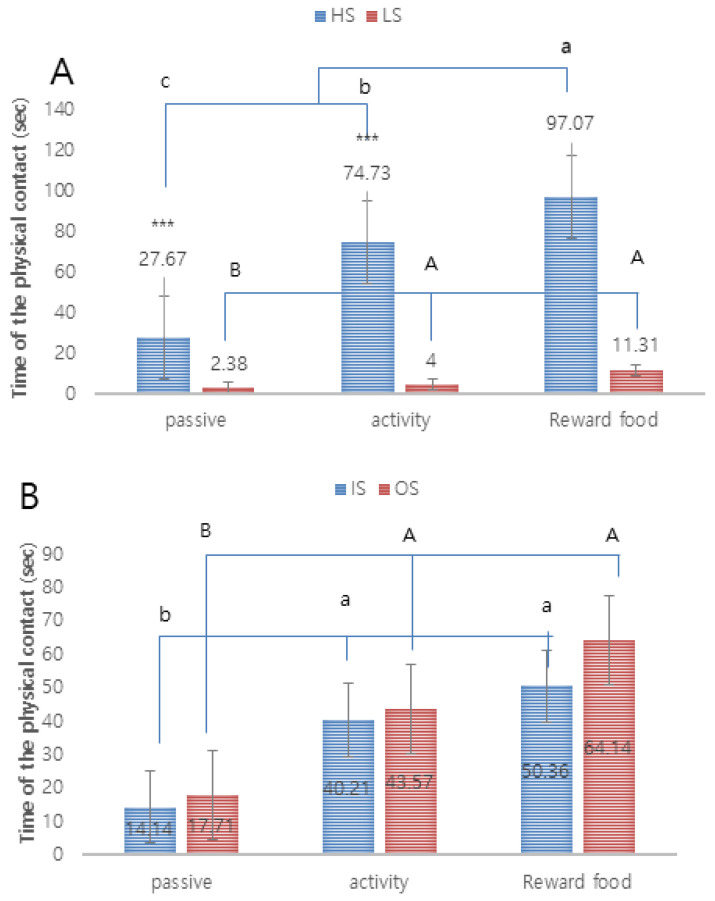
Behavioral changes in dogs by stages in the 9 social behavior test categories. (**A**): Comparison between HS group and LS group. A,B mean a significant difference for each stage within the same LS group. a–c means the significant difference by step within the same HS group (*p* < 0.05). Levels of significance: *** *p*<0.001. (**B**): Comparison between IS group and OS group. A,B mean a significant difference in stages within the same OS group. A,B mean a significant difference in stages within the same OS group. a,b means a significant difference by step within the same IS group (*p* < 0.05).

In a comparison between the HS and LS groups, the HS group showed 100% success in all social behavior categories, with the exception of close to trainer within 1 m of passive phase (93%), whereas the LS group showed a 100% success rate for physical contact with the trainer of passive, active, and food reinforcement phase (Figure 2A). There was no significant difference in the success rates of the behavioral categories in the IS and OS groups (Figure 2B). In addition, in the stepwise comparison of contact, which is an important element of sociality, the HS group showed significant differences in physical contact with the trainer of passive phase (27.67 ± 15.00 s), physical contact with the trainer of active phase (74.73 ± 35.02 s), and physical contact with the trainer of food reinforcement phase (97.07 ± 22.33 s) (*p* < 0.001). The LS group showed a significant difference between physical contact with the trainer of the passive phase (2.38 ± 4.54 s) and physical contact with the trainer of the food reinforcement phase (11.31 ± 11.57 s) (Figure 3A). There was no significant difference between groups in the stepwise comparison of contact between the IS and OS groups in the behavioral categories (Figure 2B). However, physical contact with the trainer of passive and active phase (*p* < 0.5), and physical contact with the trainer of passive and food reinforcement phase (*p* < 0.01) showed significant differences in the IS group, while physical contact with the trainer of passive and active phase (*p* < 0.01), physical contact with the trainer of active and food reinforcement phase (*p* < 0.001), and physical contact with the trainer of passive and food reinforcement phase (*p* < 0.001) showed significant differences in the OS group (Figure 3B).

## 4. Discussion

In this study, the sociality level of dogs was tested by examining changes in sociality in human-to-dog communication behavioral tasks via food reinforcement. Previous studies have shown that dogs trained using the negative reinforcement method exhibited stress, compared with dogs trained using positive reinforcement techniques [18]. Dogs demonstrate stress and stress-related behaviors in various ways, including a low posture, licking, and yawning [18,24]. In this study, the social training of dogs via food reinforcement was much more effective in both the HS and LS groups. In particular, the HS group showed a much higher increase in social behavior compared with the LS group, which indicated more effective reinforcement in interactions with people. Moreover, being able to confirm the level of sociality provides important clues about the dog’s behavior when coming in contact with strangers. This was evidenced by the longer contact latency time in the HS than LS group. In addition, the gaze time of the dog at the trainer also provides some indication of the influence of the sociality of the dog [6].

In contrast, differences between the IS and OS groups were not found, except in terms of the ATC, which deviated from our expectations. This result suggests that staying outdoors may not necessarily contribute to LS; i.e., an owner may not have to stay indoors to accommodate the dog, but more attention should be given to this condition. A family of dogs in a particular household generally live together (outside or inside the home); however, our results showed that the relationship between the owner and dog is more important than relationships with other dogs. Additionally, it can be difficult to detect social differences. For example, a dog may spend much of time with its owner but, when outside, may spend most of its time sleeping, as opposed to interacting with other animals or humans.

Despite these results, it cannot be concluded that there is no difference in sociality between dogs living outdoors and dogs living indoors. Because, first, the sample size is too small to reach a formal conclusion as a result of this study. Second, the meaning of staying with the owner indoors allows you to learn more about the dog’s daily life, and it is easy to grasp the dog’s behavior patterns. In addition, the communication time between the owner and the dog is more likely to be longer than that of outdoor dogs. However, the results of this study show the possibility that the sociality of dogs living outdoors may vary depending on the degree of communication with the owner. Therefore, it is thought that further research is needed in the future.

Barrera et al. [14] found that shelter dogs stayed near the experimenter longer than companion dogs, and that companion dogs remained near the door more frequently than shelter dogs. However, in our study, the time spent in close proximity to the experimenter was much longer in the LS than HS group. This may be because shelter dogs miss interacting with people in a limited space. Our study was conducted in companion dogs; thus, the HS group likely consisted of dogs that were freely active, without strangers, or with few strangers among them on a general basis.

In the human communication behavior task of this study, physical contact with the trainer of food reinforcement phase (*p* < 0.001) was significantly longer than physical contact with the trainer of the passive and active phase in the HS group, and RPC (*p* < 0.05) was shorter from physical contact with the trainer of passive phase in the LS group. The level of sociality in both groups increased according to the steps but was significantly lower in the LS than HS group. This is an important indicator of the level of sociality. Also, the execution of orders from strangers by the dogs shows an important level of interaction with people other than those within the dog’s household. Thus, considering that the human–dog interaction is a very important factor for the dog to be a successful companion, dog sociality can be regarded as a predictor of companionability. Dogs with low social skills can improve their social skills through various training. One of them is effective in instilling good memories of what the dog is on guard against (unfamiliar animals or people, etc.). At this time, positive reinforcement education plays an important role in effective training. For example, in a dog that is wary of strangers, giving a snack every time a stranger is seen (instilling a memory that a good thing happens) will reduce the dog’s vigilance against strangers. Through this, it is possible to gradually form bonds with strangers through repeated education. This continuous owner’s effort plays an important role in enhancing the dog’s sociality. The important thing at this time is that the owner must understand the dog’s signals or behaviors and respond appropriately.

Taken together, the results of our suggest show that dogs with high social skills are more capable of adapting to new environments than dogs with low social skills and that the sociality of dogs is closely related to their ability to communicate with people and their training.

## 5. Conclusions

This study evaluated the sociality of dogs living indoors and dogs living outdoors and classified them into high and low social groups. The results showed the change in the dog’s behavior, according to the level of the dog’s sociality through the communication behavior task. Specifically, the HS group showed a greater increase in social behavior according to stage compared with the LS group. In addition, in the communication behavior task with humans, the group with HS showed much more positive behavioral changes than the group with LS. This is an important result of sociality level, as positive communication behavior and training results indicate the ability of the dog to better adapt and interact with its environment and others, especially members of its human family

## Figures and Tables

**Table 1:** Categories for evaluating social behavior in dogs.

	Passive Phase (P)	Active Phase (A)	Food Reinforcement (R)
Contact latency (CL)	The time it took for the dog to first approach the trainer (s)		
Time close to trainer (TC)	The time the dog approaches the trainer within 1 m distance (s)		
Physical contact (PC)	Physical contact time between dog and trainer (s)		

**Table 2:** Results of the nine social behavior test categories.

Group	Passive (s)			Activity (s)			Food Enrichment (s)		
	PCL	PTC	PPC	ACL	ATC	APC	RCL	RTC	RPC
HS	7.33 ^b^ ± 3.87	6.60 ^b^ ± 4.98	27.67 ^a^ ± 15.00	6.13 ^b^ ± 5.17	6.33 ^b^ ± 6.96	74.73 ^a^ ± 35.02	7.73 ^b^ ± 3.84	4.53 ^c^ ± 12.85	97.07 ^a^ ± 22.33
LS	20.38 ^a^ ± 31.39	24.75 ^a^ ± 19.05	2.58 ^b^ ± 4.54	6.69 ^ab^ ± 9.72	15.85 ^a^ ± 19.49	4.00 ^bc^ ± 7.11	14.62 ^a^ ± 12.24	12.85 ^b^ ± 12.26	11.31 ^ab^ ± 11.57
P	0.121	0.004	0.000	0.848	0.025	0.000	0.049	0.017	0.000
IS	19.79 ± 29.78	16.14 ± 20.05	14.14 ± 18.25	7.14 ^bc^ ± 9.42	15.36 ^ab^ ± 13.98	40.21 ^a^ ± 45.86	14.00 ^b^ ± 11.77	10.57 ^c^ ± 12.76	50.36 ^a^ ± 51.75
OS	7.00 ^b^ ± 6.45	12.14 ^b^ ± 10.41	17.71 ^a^ ± 16.20	5.64 ^b^ ± 5.12	6.14 ^b^ ± 5.57	43.57 ^a^ ± 44.02	7.86 ^b^ ± 4.57	6.21 ^c^ ± 3.64	64.14 ^a^ ± 42.66
P	0.129	0.513	0.589	0.605	0.030	0.845	0.080	0.230	0.449

^a–c^ Least-square means with different superscripts in the same row significantly differ (*p* < 0.05). PCL, contact latency of passive phase; PTC, time close to trainer of passive phase; PPC, physical contact time of passive phase; ACL, contact latency of active phase; ATC, time close to trainer of active phase; APC, physical contact with the trainer of active phase; RCL, contact latency of food reinforcement phase; RTC, time close to trainer of food reinforcement phase; RPC, physical contact with the trainer of food reinforcement phase.
